# Comprehensive proteomic profiling of intestinal tissues in patients with ulcerative colitis

**DOI:** 10.3389/fmed.2025.1537168

**Published:** 2025-06-13

**Authors:** Huiling Wang, Qi Xie, Yi Xie, Weiguang Luo

**Affiliations:** ^1^Department of Clinical Laboratory, Henan Provincial People's Hospital, People's Hospital of Zhengzhou University, Zhengzhou, China; ^2^Department of Pathology, Henan Provincial People's Hospital, People's Hospital of Zhengzhou University, Zhengzhou, China; ^3^Department of Gastrointestinal Surgery, Henan Provincial People's Hospital, People's Hospital of Zhengzhou University, Zhengzhou, China

**Keywords:** ulcerative colitis, proteomics, LC–MS/MS, intestinal tissues, inflammation

## Abstract

**Introduction:**

Ulcerative colitis (UC) is a chronic inflammatory bowel disease characterized by persistent inflammation of the colonic mucosa. This condition can significantly affect the quality of life of those affected. While UC is common, its underlying mechanisms are not yet fully understood, highlighting the need for a comprehensive proteomic analysis of intestinal tissues to identify potential biological changes associated with the disease. This study aimed to investigate the proteomic differences in the intestinal tissues of patients with UC and healthy individuals using high-throughput liquid chromatography–tandem mass spectrometry (LC–MS/MS) and bioinformatics methods.

**Methods:**

The study employed a comprehensive proteomic analysis using LC–MS/MS to identify protein expression differences in intestinal tissues from five patients with UC versus five healthy controls. Subsequent bioinformatics analyses, including Gene Ontology (GO) and Kyoto Encyclopedia of Genes and Genomes (KEGG) pathway analyses, elucidated altered biological processes.

**Results:**

We identified 194 upregulated and 323 downregulated proteins in the tissues of patients with UC, indicating a significant difference in protein expression. GO analysis revealed that the upregulated proteins were mainly involved in immune responses and metabolic processes, while the downregulated proteins were associated with organic and cellular metabolism. Additionally, KEGG pathway analysis showed that upregulated proteins were enriched in pathways related to ribosomes and phagosomes, whereas downregulated proteins were primarily linked to oxidative phosphorylation, thermogenesis, and the citric acid cycle, pointing to substantial changes in cellular energy metabolism. Protein–protein interaction (PPI) network analysis identified several key nodes, particularly those connected to ribosomal and phagocytic functions, which may play significant roles in the pathophysiology of UC.

**Conclusion:**

This study offers new insights into the biological mechanisms underlying UC and lays the foundation for future therapeutic strategies targeting these proteomic changes. Further experimental validation and clinical investigations are necessary to uncover additional mechanisms of UC and to facilitate the development of effective treatments.

## 1 Introduction

Ulcerative Colitis (UC) is a chronic inflammatory condition of the colon, characterized by periods of flare-ups and remission of mucosal inflammation that typically begins in the rectum and extends to the proximal colon (1) Recently, there has been a significant rise in the global incidence and prevalence of UC. Current estimates indicate that approximately 0.5 to 1% of the population in Western countries is affected, and its incidence is also increasing in developing nations (2). UC substantially diminishes the quality of life of patients, causing symptoms such as abdominal pain, diarrhea, and fatigue (3), which can lead to serious complications, including an increased risk of colorectal cancer (4).

The etiology and pathogenesis of UC remain incompletely understood, and some patients ultimately require surgical intervention, including total colectomy (5). Current clinical treatments mainly involve anti-inflammatory medications, immunosuppressants, and biologics; however, these methods often produce inconsistent responses in patients and can be associated with considerable side effects (6). Therefore, there is an urgent need for innovative diagnostic and therapeutic approaches to enhance our understanding of UC and improve patient outcomes (7).

Proteomics involves the large-scale identification of proteins and often encompasses the study of all proteins expressed by an organism, known as the proteome (8). The advent of “omics” technologies has enabled high-throughput analysis of genes, proteins, and metabolites, along with their expression levels. Unlike genomics or transcriptomics, which focus on static genetic blueprints or transient RNA profiles, proteomics directly interrogates the dynamic, functional molecules that drive cellular processes (9, 10). This unique capability positions proteomics as an indispensable approach for understanding disease mechanisms, identifying therapeutic targets, and advancing personalized medicine.

Several studies have highlighted specific protein changes linked to UC (11, 12), such as the upregulation of pro-inflammatory cytokines and the downregulation of proteins critical for maintaining epithelial barrier function (13). However, despite these insights, there remains a considerable gap in our understanding of the complete proteomic landscape of UC, particularly in identifying potential biomarkers and therapeutic targets. The primary objective of our study was to identify proteins differentially expressed in UC tissues compared to healthy controls. By characterizing these proteins, we aimed to uncover their potential biological roles in UC, with a particular focus on immune responses and metabolic pathways related to inflammation. This study aims not only to advance the current literature on UC but also to identify novel biomarkers that may guide future therapeutic strategies, ultimately improving patient outcomes and quality of life.

## 2 Materials and methods

### 2.1 Protein extraction

Formalin-fixed, paraffin-embedded (FFPE) samples were extracted using the BioGnostics FFPE Total Protein Extraction Kit (C500058-0050). The protein concentration was determined using a BCA protein assay kit. This research was approved by the Ethics Committee of Henan Provincial People’s Hospital (No. 202162). UC tissues of five patients (2 males, 3 females, average age: 41 ± 3.2) and pathologically confirmed normal colon tissues of five healthy individuals (2 males, 3 females, average age: 38 ± 2.2) undergoing surgical resection at the Department of Gastrointestinal Surgery of Henan Provincial People’s Hospital were collected. All of the samples and data were collected after written informed consent was provided by the participants. The management and publication of patient information in this research was strictly in accordance with the Declaration of Helsinki, including confidentiality and anonymity; data were de-identified before analysis.

### 2.2 RNA preparation, Q-PCR for mRNA

Total RNA was extracted from colon biopsies using Trizol Reagent (Invitrogen, Carlsbad, CA, USA).The Transcriptor First Stand cDNA Synthesis Kit (Roche) and the Fast Start Universal SYBR Green Master (Roche) were used to confirm the mRNA expression changes. The expression of each target mRNA was calculated, respectively, relative to *β*-Actin. A comparative threshold cycle method was used to compare each condition with controls. The data are shown as means ± SD. Statistical analyses were performed using GraphPad Prism 8. Statistical comparisons between groups were performed using the Student’s t-test, with a significance threshold of *p* < 0.05.

The primers were as follows: PRTN3: Forword: AACTACGACGCGGAGAACAAA, Reverse: CGAGGGACGAAAGTGCAAATG; MPO: Forword: GGGAGCGATTGTTTGAGCA, Reverse: TGTTGGGCGTGCCATACTG; S100A9: Forword: ACCATCATCAACACCTTCCACC, Reverse: GTTAGCCTCGCCATCAGCA; DEFA3 Forword: ATTGCAGCGGACATCCCA, Reverse: GCAGCAGAATGCCCAGAGTC; RCN3: Forword: CTTCTGTTGCTACTGAGGCACG, Reverse: TCCTCTGGGGTGAGTTGGTC; HMGCS2: Forword: AGGTCTACTTCCCAGCCCAATA, Reverse: GGACTGAGCAGAAGCCCATAC; CDH17: Forword: CCGGAAGTCCATATCGGGTAC, Reverse: GCCTGGGAGGGTTGTCATT; VIL1: Forword: AAGCAGTACCCACCAAGCACA, Reverse: CCCACTCCCATCATCTACCATC; HADH: Forword: CATAGCGACCAGCACGGAT, Reverse: AAATGGAGGCCAGCGAATC; LIMA1: Forword: ACAAGGATCTATGGGCAAGCA, Reverse: CCAGGACACCCACCTTAGCA.

### 2.3 Trypsin digestion

For digestion, the protein solution was reduced with 5 mM dithiothreitol for 30 min at 56°C and then alkylated with 11 mM iodoacetamide for 15 min at room temperature in the dark. The protein sample was subsequently diluted with 100 mM TEAB to achieve a urea concentration of less than 2 M. Finally, trypsin was added at a 1:50 trypsin-to-protein mass ratio for the initial overnight digestion, followed by a second digestion for 4 h at a 1:100 trypsin-to-protein mass ratio. The protein concentration was measured using the BCA kit (Beyotime Biotechnology, P0012) before proceeding to the next experiment.

### 2.4 LC–MS/MS analysis

The tryptic peptides were dissolved in 0.1% formic acid (solvent A) and directly loaded onto a homemade reversed-phase analytical column (15-cm length, 75 μm i.d, C18 solid-phase extraction membrane 3 M, 2215-C18). The gradient comprised an increase from 6 to 23% solvent B (0.1% formic acid in 98% acetonitrile) over 26 min, from 23 to 35% over 8 min, and then increasing to 80% over 3 min, holding at 80% for the final 3 min, all at a constant flow rate of 400 nL/min using an EASY-nLC 1,000 UPLC system.

The peptides were subjected to nano-spray ionization (NSI), followed by tandem mass spectrometry (MS/MS) in the Orbitrap Exploris 480 (Thermo), coupled online to UPLC. The applied electrospray voltage was 2.0 kV. The m/z scan range was 350–1800 for the full scan, with intact peptides detected in the Orbitrap at a resolution of 70,000. Peptides were then selected for MS/MS using an NCE setting of 28, with the fragments detected in the Orbitrap at a resolution of 17,500. A data-dependent procedure alternated between one MS scan followed by 20 MS/MS scans with a 15.0 s dynamic exclusion. The automatic gain control (AGC) was set to 5E4, and the first fixed mass was set to 100 m/z.

### 2.5 Database search

The resulting MS/MS data were processed using the Proteome Discoverer search engine (version 1.5.2.8). Tandem mass spectra were searched against the Human database (*Homo sapiens* 9,606) concatenated with a reverse decoy database. Trypsin/P was used as the cleavage enzyme, allowing for up to two missing cleavages. The mass tolerance for precursor ions was set to 20 ppm for the first search and 5 ppm for the main search. The mass tolerance for the fragment ions was set to 0.02 Da. Carbamidomethyl on Cysteine (Cys) was specified as a fixed modification, and oxidation on methionine (Met) was specified as a variable modification. The false discovery rate (FDR) was adjusted to < 1%, and the minimum score for the peptides was set to > 40. A total of 3,603 proteins were confidently identified using a threshold of >1 unique peptide (Supplementary Table S1). 2,745 proteins were preserved following removing of those exhibiting more than 50% missing values across all samples (Supplementary Table S2).Residual missing values were retained without imputation and carried forward for subsequent analyses. Proteome intensities were normalized by subtracting the median intensity of each sample.

### 2.6 Bioinformatics analysis

Gene Ontology (GO) annotation of the resulting proteome was performed using the UniProt-GOA database (http://www.ebi.ac.uk/GOA/). Proteins were classified according to GO annotation in three categories: biological processes, cellular components, and molecular functions. The subcellular localization of the proteins obtained by mass spectrometry was predicted using Wolfpsort (http://wolfpsort.seq.cbrc.jp/). The Kyoto Encyclopedia of Genes and Genomes (KEGG) database was used to annotate the protein pathways. First, the KEGG online service tool KAAS was used to annotate the protein KEGG database descriptions. The KEGG online service tool KEGG mapper was used to map the annotation results onto the KEGG pathway database. For functional enrichment analysis of GO and KEGG, a two-tailed Fisher’s exact test was used to assess the enrichment of differentially expressed proteins against all identified proteins. The subcellular localization analysis uses the UniProt subcellular localization database. GO and KEGG pathways with a corrected *p*-value <0.05 were considered significant. The physical and functional interaction networks of all differentially expressed proteins were analyzed using the STRING database (version 11.5, www.cn.string-db.org/) and visualized using Cytoscape (version 3.9.1, www.cytoscape.org). Cluster analysis was analyzed using the MCODE. The GO and KEGG enrichment tests and graphs were conducted using “Hmisc” and “ggplot2” R packages.

## 3 Results

### 3.1 Quantitative signatures of altered proteins

**Figure 1:**
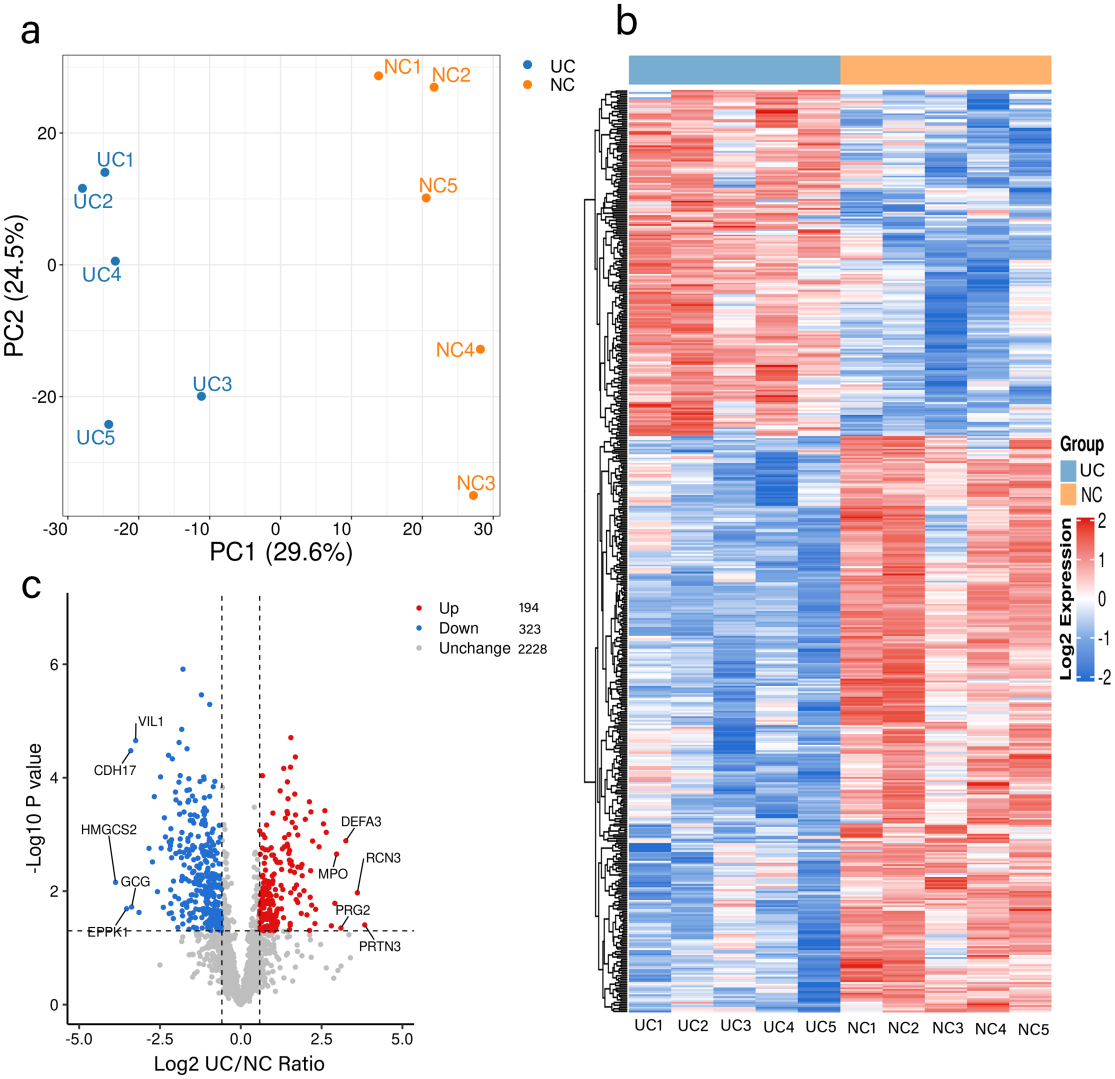
Quantitative signatures of altered proteins in surgical specimens of patients with UC. **(a)** Principal component analysis was performed based on the log2-transformed intensity of proteins identified in the three replicates. **(b)** Hierarchical cluster analysis of the proteins identified in the UC and NC groups. **(c)** Volcano plot graphs representing the comparative analysis between the UC and NC groups. The X-axes correspond to the ratio of intensities in log2 scale for each protein in the two groups, and the Y-axes are -log10-transformed adjusted *p*-values (from a two-sample t-test with FDR correction). Blue dots and orange dots represent proteins down-regulated and up-regulated in UC tissues, respectively. UC: Ulcerative Colitis group, NC: Control group.

To ascertain whether the tissue samples could distinguish between normal and UC intestinal tissues, a quantitative analysis of the proteomic data was conducted. 2,745 proteins (Supplementary Table S2) were subjected to quantitative analysis. Principal components analysis (PCA) was performed to distinctly categorize the proteomic outcomes of normal (yellow dots) and UC tissues (blue dots) (Figure 1a). The heat map results indicated that the gene expression in UC samples was markedly different from that in the control group (Figure 1b). Proteins exhibiting fold-change (FC) ratios greater than 1.5 for the upregulation threshold and less than 0.67 for the down-regulation threshold, along with a *p*-value of less than 0.05, were considered significantly different between normal and UC intestinal tissues and earmarked for further analysis (Figure 1c). In comparison to the control group, 194 proteins were upregulated in UC tissues (red), and 323 proteins were downregulated in UC tissues (blue) (Figure 1c; Supplementary Table S3).

### 3.2 Functional classification and subcellular localization of altered proteins in control and UC tissues

**Figure 2:**
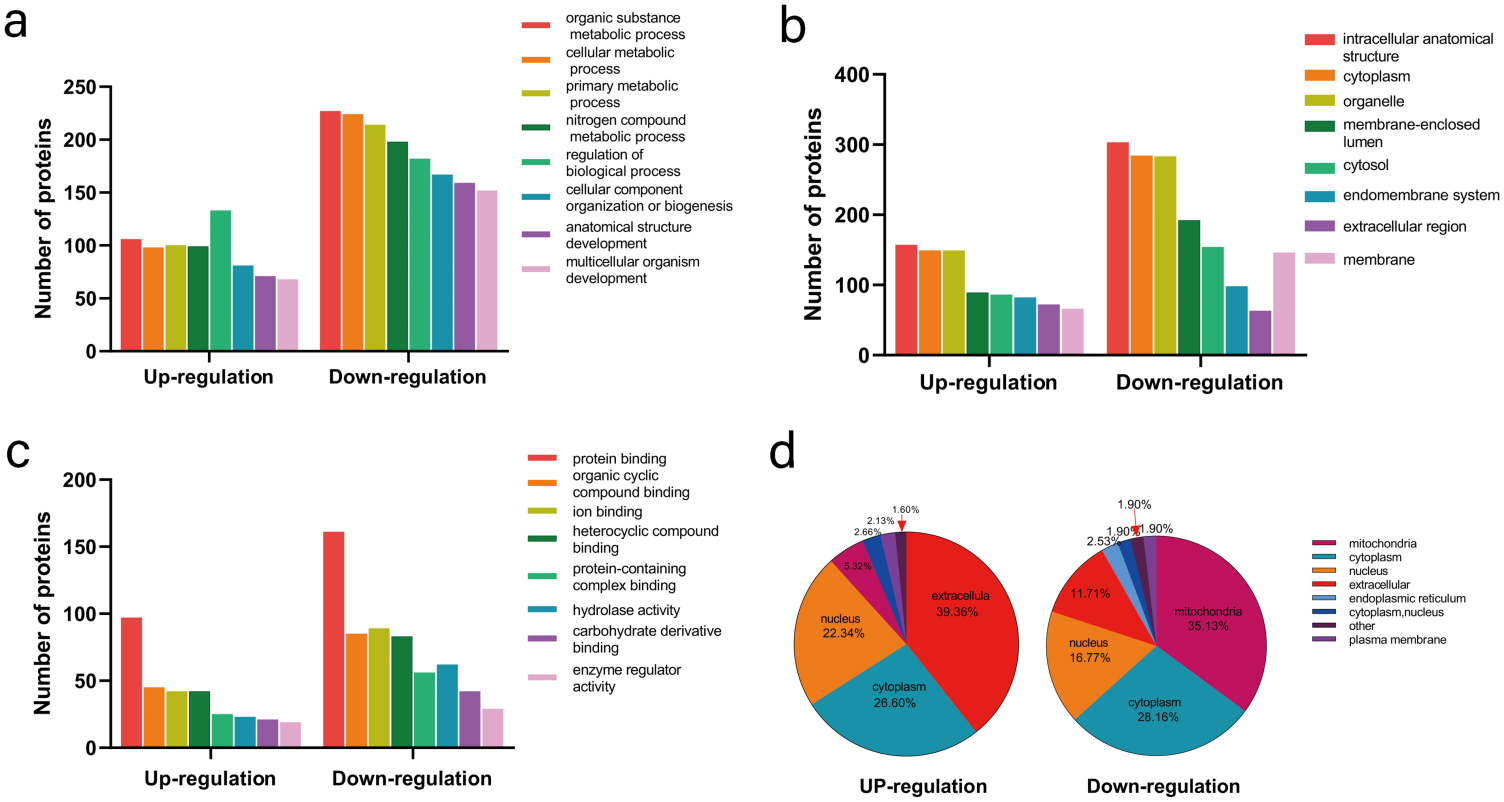
Functional classification and subcellular localization of altered proteins in control and UC tissues. **(a)** Classification of altered proteins according to their biological processes. **(b)** Classification of altered proteins based on their cellular components. **(c)** Classification of altered proteins according to their molecular functions. **(d)** Analysis of the subcellular localization of the altered proteins.

To further explore the potential functions of the modified proteins in UC, all differentially expressed proteins were categorized using GO functional classification based on their biological processes, cellular compositions, and molecular functions. Among the biological processes, a significant proportion of the upregulated proteins was associated with the regulation of biological processes (133/194), organic substance metabolic processes (106/194), and primary metabolic processes (100/194) in UC tissues. In contrast, the downregulated proteins were primarily linked to organic substance metabolic processes (227/323), cellular metabolic processes (224/323), and primary metabolic processes (214/323) (Figure 2a; Supplementary Table S3). There were no discernible differences between the upregulated and downregulated proteins regarding cellular components and molecular functions (Figures 2b,c; Supplementary Table S4). These findings suggest that the biological functions of proteins may be altered in UC, potentially affecting various biological processes. The subcellular localization of all modified proteins was also examined. As shown in (Figure 2d; Supplementary Table S5), the altered proteins were broadly distributed across various cellular components. The upregulated proteins were predominantly located in the extracellular space (39.36%), whereas the downregulated proteins were primarily found in the mitochondria (35.13%).

### 3.3 Functional enrichment analysis of altered proteins in UC

**Figure 3:**
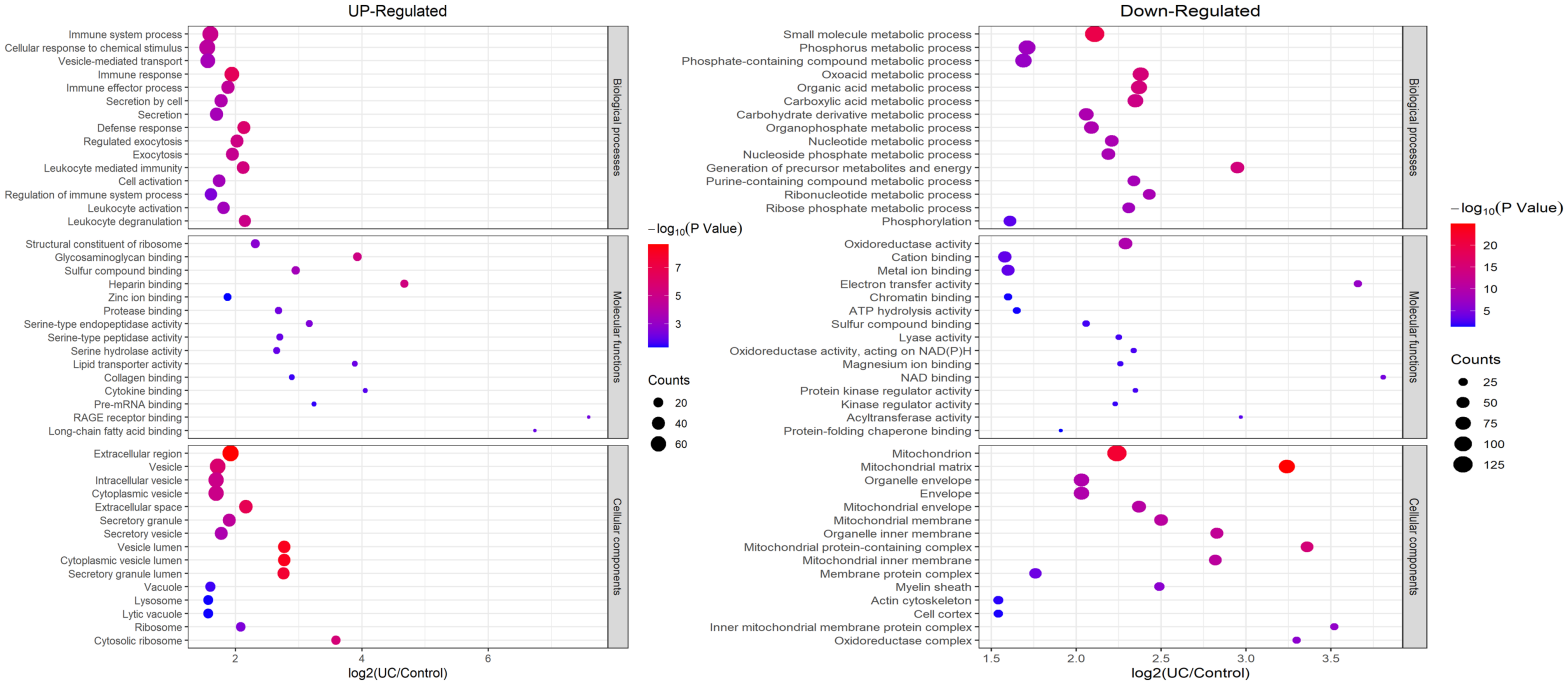
Enrichment analysis of the altered proteins based on GO in UC tissue. (left) Up-regulated; (right) Down-regulated.

**Figure 4:**
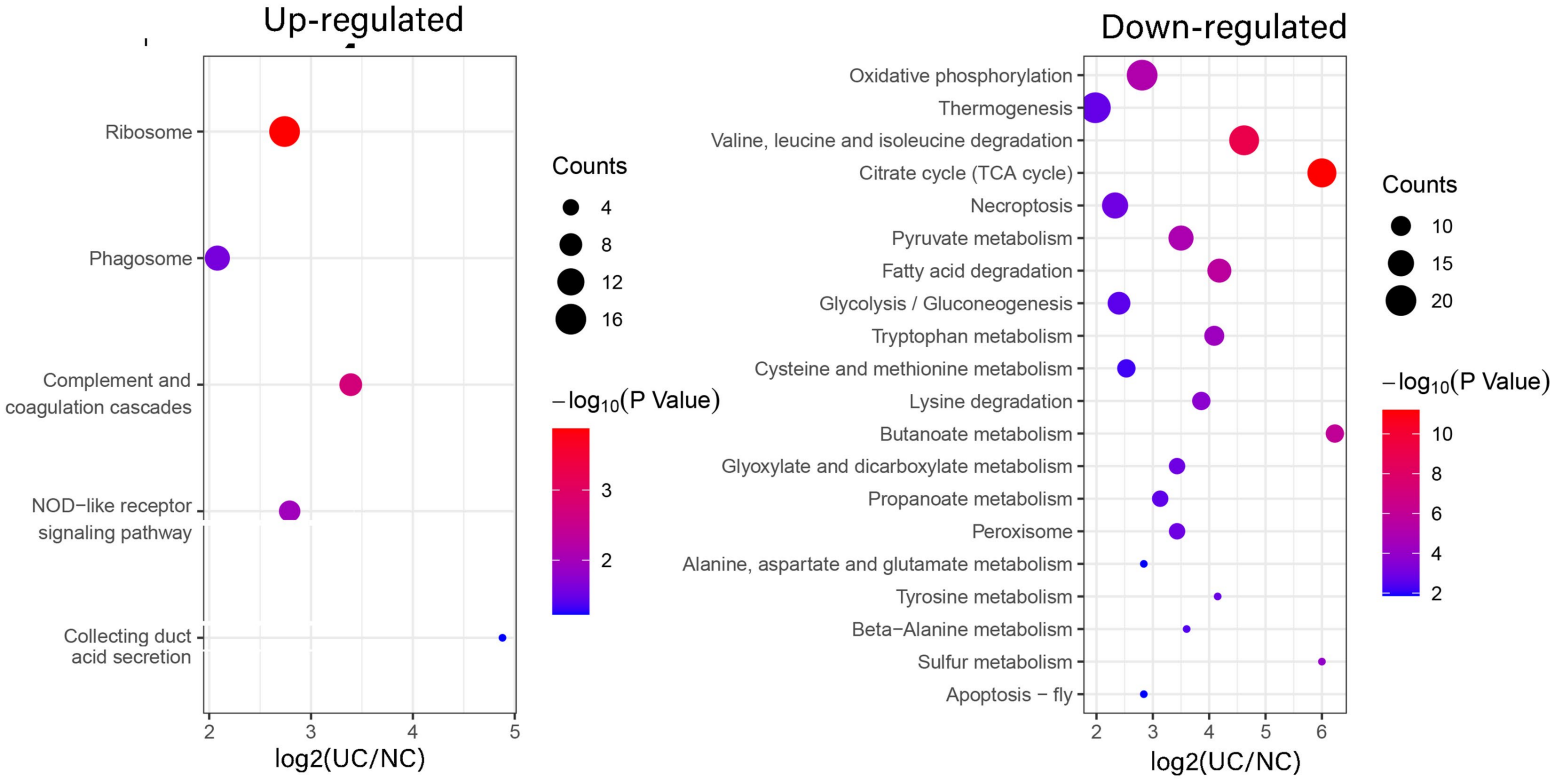
Enrichment analysis of the altered proteins in UC tissue according to the KEGG pathway. (left) Up-regulated; (right) Down-regulated.

To elucidate the favored target protein types of the altered proteins, a functional enrichment analysis of the acquired proteins was conducted using GO and KEGG pathways. In the biological process analysis, upregulated proteins were enriched in immune system processes, cellular responses to chemical stimuli, and immune responses. Upregulated proteins involved in immune responses suggest an exacerbated inflammatory state in UC. These findings align with the chronic inflammation observed in patients with UC. Conversely, the majority of downregulated proteins were enriched in small molecule and phosphorus metabolic processes. In terms of molecular functions, the upregulated proteins were significantly enriched in structural constituents of ribosomes, glycosaminoglycan binding, and sulfur compound binding, while the downregulated proteins were enriched in cation binding and oxidoreductase activity. In the enrichment analysis of cellular components, the upregulated proteins were predominantly enriched in vesicles and the extracellular region, whereas the downregulated proteins were enriched in mitochondria, mitochondrial matrices, and organelle envelopes (Figure 3; Supplementary Table S6). The downregulation of mitochondrial proteins points to impaired energy production and cellular homeostasis, which may contribute to tissue damage in UC. KEGG pathway analysis revealed that ribosomes and phagosomes were enriched among the upregulated proteins, while pathways enriched among the downregulated proteins included oxidative phosphorylation, thermogenesis, and the citrate cycle (TCA cycle) (Figure 4; Supplementary Table S7). The upregulation of ribosomal proteins suggests increased protein synthesis, potentially as a compensatory mechanism during inflammation. Phagosome-related proteins highlight the role of immune cell activity in UC pathogenesis. Downregulated proteins associated with oxidative phosphorylation and the TCA cycle indicate a shift in cellular energy metabolism. This metabolic reprogramming may reflect cellular adaptation to chronic inflammation and energy depletion.

### 3.4 Protein–protein interactions network of altered proteins

**Figure 5:**
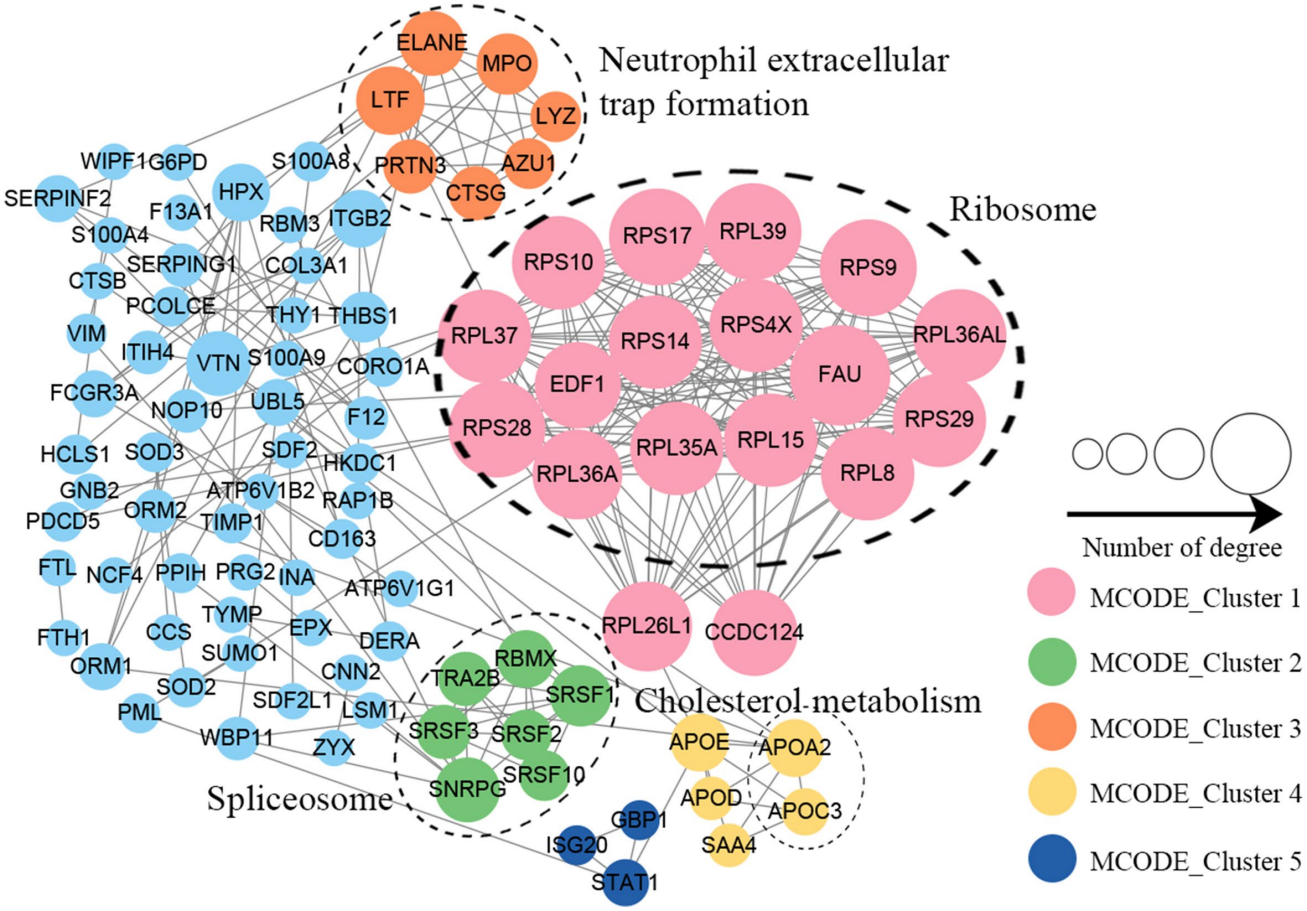
Protein–protein interaction network of upregulated proteins in UC tissues.

**Figure 6:**
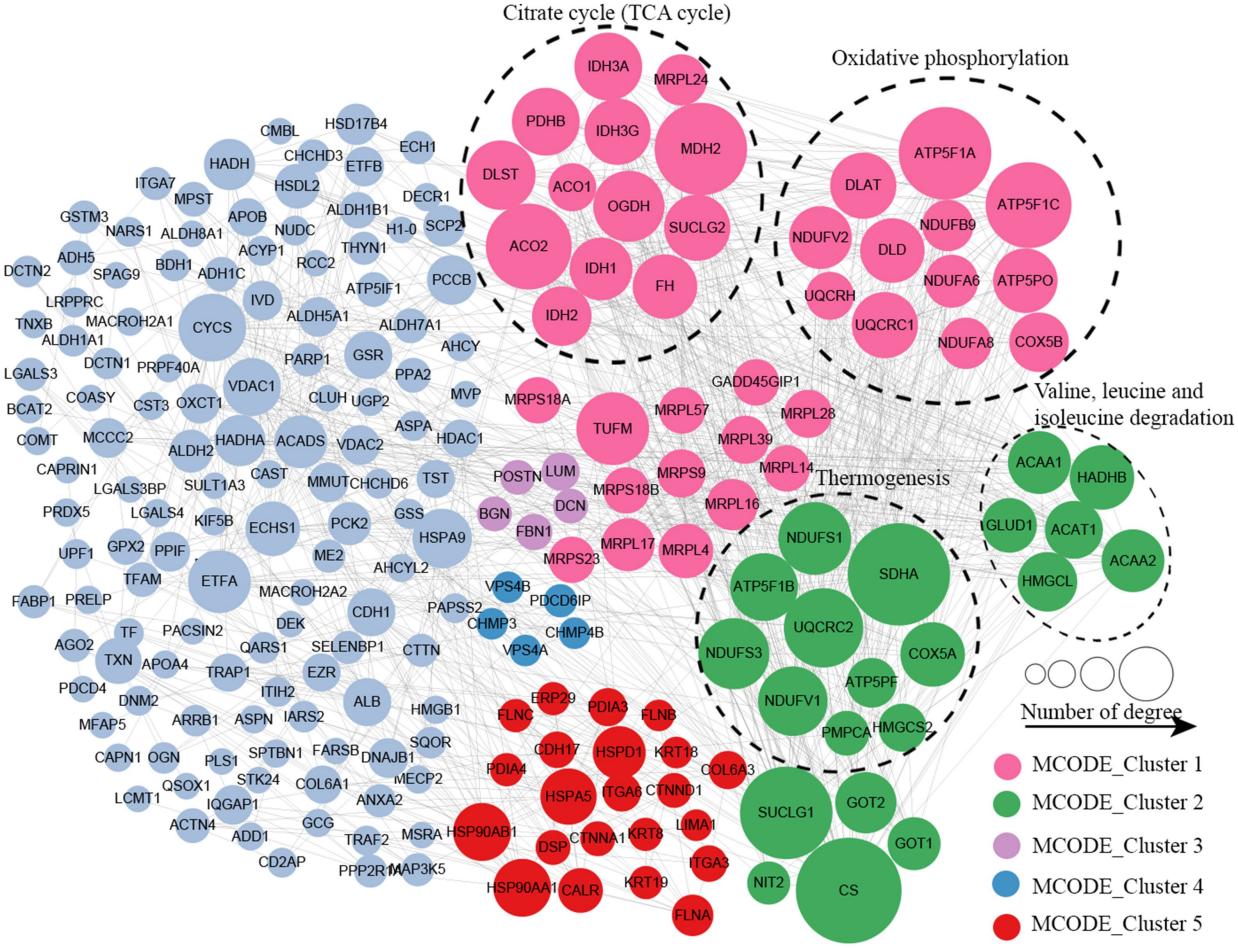
Protein–protein interaction network of downregulated proteins in UC tissues.

Protein–protein interaction (PPI) network analysis was conducted to identify the principal nodes and significant biological processes among the differentially expressed proteins. As illustrated in Figure 5 and Supplementary Table S8, 95 nodes and 275 interactions of the upregulated proteins were identified in the PPI network database. Using Cytoscape software and Minimal Common Oncology Data Elements (MCODE), several highly associated protein subnetworks were identified. In accordance with the KEGG enrichment results, proteins associated with ribosomes were distinctly upregulated in UC, and proteins related to neutrophil extracellular trap formation and spliceosome were also found to be elevated. In contrast, 250 nodes and 1,180 interactions were discovered among the downregulated proteins in the PPI network database, providing a comprehensive overview of the pathways implicated by the differentially expressed proteins. Several proteins linked to TCA cycle, oxidative phosphorylation, and thermogenesis, as well as valine, leucine, and isoleucine degradation were also downregulated (Figure 6; Supplementary Table S9).

### 3.5 Verification of altered proteins in UC

**Figure 7:**
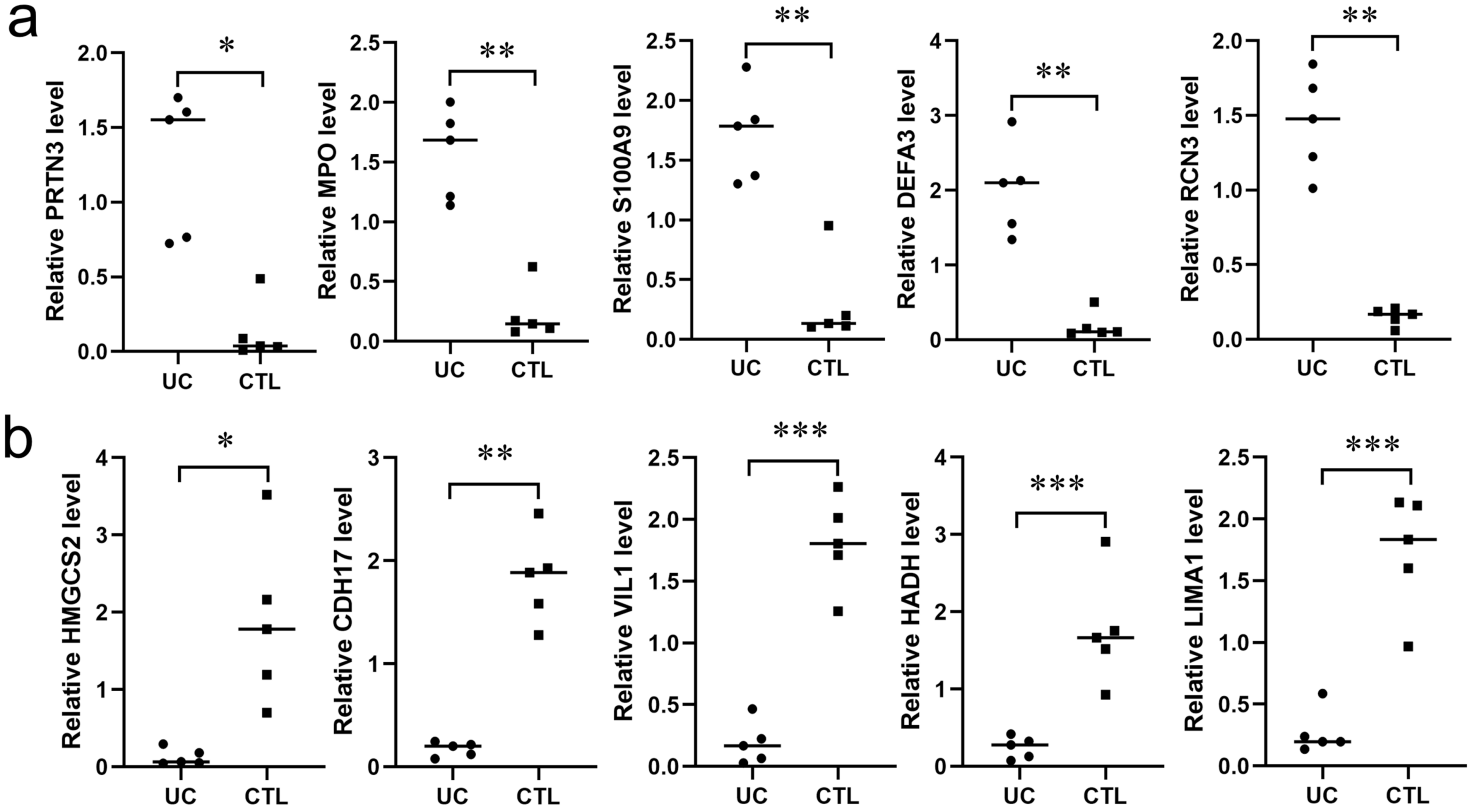
Verification of altered proteins in UC tissues. **(a)** The mRNA expression levels of five representative proteins (PRTN3, MPO, S100A9, DEFA3, RCN3) among the up-regulated proteins (*n* = 5). **(b)** The mRNA expression levels of five representative proteins (HMGCS2, CDH17, VIL1, HADA, LIMA1) among the down-regulated proteins (*n* = 5). * *p* < 0.05, ** *p* < 0.01, *** *p* < 0.001.

To validate the altered proteins in UC, we selected five proteins each from the top 10 upregulated and downregulated candidates and measured their corresponding mRNA levels using RT-PCR. As shown in Figure 7, the mRNA levels of PRTN3, MPO,S100A9,DEFA3,RCN3 were significantly elevated in UC tissues, whereas those of HMGCS2, CDH17, VIL1, HADA, LIMA1 were markedly reduced, consistent with the proteomic findings.

## 4 Discussion

UC is a chronic inflammatory bowel disease characterized by inflammation and ulceration of the colonic mucosa. The etiology of UC remains largely unknown; however, it is believed to involve a complex interplay between genetic predisposition, environmental factors, and immune dysregulation. The chronic nature of UC not only affects patients’ physical health but also significantly diminishes their quality of life, highlighting the urgent need for a deeper understanding of its underlying mechanisms. Moreover, individuals with UC have a heightened risk of developing colorectal cancer, particularly after living with the disease for an extended period, underscoring the need for continued research in this area (14).

In this study, we aimed to investigate the proteomic alterations in the intestinal tissues of patients with UC compared to healthy controls. Using advanced techniques such as liquid chromatography–tandem mass spectrometry (LC–MS/MS) combined with bioinformatics analyses, we identified 194 upregulated and 323 downregulated proteins in UC tissues. Our findings revealed significant dysregulation of proteins associated with immune response and metabolic pathways, suggesting that these alterations may play a pivotal role in the pathogenesis of UC. Pathway enrichment analysis indicated the involvement of ribosomal and phagocytic processes in disease progression, providing valuable insights into the cellular mechanisms underlying UC. These results not only contribute to the understanding of UC’s biological complexity but also pave the way for identifying potential biomarkers and therapeutic targets for future treatment.

Recent advances in multi-omics approaches have significantly enhanced our understanding of the molecular mechanisms underlying inflammatory bowel diseases (IBD), including UC. For instance, Schniers et al. conducted an in-depth proteomic analysis of patients with UC, identifying key dysregulated pathways in mucosal tissues, such as oxidative stress responses and metabolic reprogramming. Their work underscores the value of proteomics in unraveling disease-specific signatures, though it also highlights the need for integrative approaches to connect proteomic findings to transcriptional regulation (15). Proteomic profiling of 17 UC patient tissues and 15 healthy controls identified 275 upregulated and 321 downregulated proteins, with the increased sample size likely contributing to the robust detection of differentially expressed proteins (15). Notably, comparative analysis revealed 35 overlapping proteins in the upregulated subset and 63 in the downregulated subset between our dataset and prior studies (Supplementary Figure S1a; Supplementary Tables S10–11). KEGG pathway enrichment analysis revealed that neutrophil extracellular trap formation and phagosomes were enriched among the up-overlap proteins, while metabolic pathways, valine, leucine, and isoleucine degradation, fatty acid degradation were enriched among the up-overlap proteins(Supplementary Figure S1b; Supplementary Table S12). These findings collectively reinforce the pivotal involvement of the aforementioned signaling pathways in both the pathogenesis and clinical progression of ulcerative colitis (UC), which warrants further mechanistic exploration through integrated multi-omics approaches and *in vivo* validation using patient-derived organoid models.The above results reinforces the reliability of our findings and suggests that prioritizing these overlapping proteins in subsequent investigations may uncover critical biomarkers for UC pathogenesis.

The significant differential protein expression observed in UC tissues offers critical insights into the molecular mechanisms underlying this complex disease. Our findings, which identified 194 upregulated and 323 downregulated proteins, suggest that immune responses and metabolic processes are central to the pathology (16). Specifically, the upregulated proteins associated with immune responses may indicate an exacerbated inflammatory state, potentially contributing to the chronic nature of the disease (17). Moreover, the downregulated proteins linked to organic and cellular metabolism highlight a potential metabolic dysfunction in patients with UC, suggesting that the disease may not only be an inflammatory disorder but also a metabolic one. This pathophysiological duality supports the hypothesis that targeted therapies addressing both inflammation and metabolic dysregulation may be beneficial for patients with UC, paving the way for novel therapeutic strategies that can enhance clinical outcomes (18).

Furthermore, our comprehensive analysis using GO and KEGG pathways revealed critical signaling pathways, including those related to ribosome function and phagocytosis, that are altered in UC. The enrichment of upregulated proteins in ribosomal pathways suggests that protein synthesis may be enhanced in response to inflammation, potentially as a compensatory mechanism to meet increased cellular demands during UC flares (19). Conversely, the downregulation of proteins involved in oxidative phosphorylation and the citric acid cycle indicates a shift in energy metabolism, possibly reflecting cells’ adaptation to chronic inflammation (20). The results of study of Schniers et al. (15) demonstrated that protein abundances involved in immune response and protein processing within the endoplasmic reticulum were significantly elevated in UC. Conversely, proteins associated with metabolic functions of nutrients, energy, steroids, xenobiotics, and carbonate showed markedly reduced abundances in UC tissues. These conclusions are consistent with our research findings.

The upregulation of ribosomal proteins observed in this study highlights the critical role of ribosomes in the pathogenesis of UC (21). Ribosomes are essential cellular machinery responsible for protein synthesis, and their dysregulation can have profound effects on cellular function, particularly in chronic inflammation and tissue repair (22). The upregulation of ribosomal proteins in UC tissues underscores their critical role in inflammation, immune activation, and metabolic stress. These findings suggest that ribosomal dysregulation is a key driver of UC pathogenesis, contributing to chronic inflammation and tissue damage. Targeting ribosomal activity or modulating protein synthesis pathways may offer novel therapeutic opportunities for patients with UC. Further research is needed to validate these findings and explore the potential of ribosomal proteins as biomarkers and therapeutic targets in UC.

The downregulation of proteins involved in the TCA cycle, oxidative phosphorylation, valine, leucine, and isoleucine degradation, and thermogenesis highlights significant metabolic dysregulation in UC. These pathways are interconnected and play critical roles in energy production, amino acid metabolism, and cellular homeostasis. Their disruption may contribute to the chronic inflammation, tissue damage, and systemic symptoms observed in patients with UC. Restoring TCA cycle activity through metabolic modulators may improve mitochondrial function (23); targeting mitochondrial function may restore energy production (24); modulating BCAA metabolism may improve metabolic homeostasis (25); and Enhancing thermogenesis may improve energy balance and reduce inflammation in UC (26).

This finding aligns with previous studies that have indicated a heightened inflammatory response in UC, wherein pro-inflammatory cytokines play a crucial role in disease progression and symptomatology. For instance, increased levels of interleukin-6 (IL-6) and other inflammatory mediators correlate with disease activity in patients with UC (27). These insights not only deepen our understanding of UC pathogenesis but also highlight potential biomarkers for disease activity and progression, which could be pivotal in developing diagnostic tools and monitoring treatment.

In addition to immune dysregulation, our findings emphasize its profound impact on cellular behavior, particularly metabolic adaptation in UC. The downregulation of proteins involved in organic and cellular metabolism may reflect a compensatory mechanism in response to chronic inflammation (28). Previous studies have demonstrated that metabolic reprogramming is a hallmark of various inflammatory diseases, including UC, where altered energy production and nutrient utilization can affect cellular proliferation and apoptosis (18, 29). Identification of these metabolic alterations can enhance our understanding of how UC affects cellular homeostasis and could lead to novel strategies aimed at restoring normal metabolic functions (30). Integrating these proteomic data with existing knowledge of gene expression and metabolic pathways may provide a comprehensive view of cellular adaptations occurring in UC, thereby facilitating the identification of biomarkers that predict disease progression.

Although this study provides significant insights into protein expression alterations in UC, certain limitations must be acknowledged. First, the small sample size may not have fully captured the heterogeneity of UC, potentially limiting the generalizability of our findings. Additionally, because our study is cross-sectional, causative relationships between changes in protein expression and disease progression cannot be inferred. Moreover, reliance on a single analytical approach, while robust, may overlook the nuances of protein interactions and post-translational modifications that play crucial roles in UC pathology. Future investigations employing longitudinal designs and integrating diverse methodologies, such as proteomics, genomics, and metabolomics, could enhance our understanding of the complex molecular landscape of UC. Thus, although the results are promising, they must be interpreted with caution, and further studies are needed to validate these findings and explore their clinical implications.

In conclusion, our comprehensive proteomic analysis revealed significant differences in protein expression between UC and normal intestinal tissues, elucidating critical pathways involved in immune response and metabolic dysregulation. The identification of 194 upregulated and 323 downregulated proteins underscores the potential of these molecular alterations as biomarkers of disease severity and targets for therapeutic intervention. Functional enrichment analyses further highlighted the importance of ribosomal and phagocytic pathways, which may contribute to the altered cellular metabolism observed in UC. These findings advance our understanding of UC’s underlying mechanisms and lay the groundwork for future studies aimed at developing targeted therapies. Continued exploration of these proteomic alterations combined with clinical validation, holds promise for transforming the management and treatment of ulcerative colitis, thereby improving patient outcomes.

## Data Availability

The original contributions presented in this study are publicly available. The mass spectrometry proteomics data have been deposited to the ProteomeXchange Consortium via the PRIDE partner repository with the dataset identifier PXD061040. This data can be found here: https://www.ebi.ac.uk/pride/login. Accession: PXD061040; Token: bMfQxC7QFFjT.
