# Supplementary material for: Comprehensive proteomic profiling of intestinal tissues in patients with ulcerative colitis
Source: Front Med (Lausanne). 2025 Jun 13;12:1537168. doi: 10.3389/fmed.2025.1537168 (PMC12203869; doi:10.3389/fmed.2025.1537168)
Supplement: Supplementary file 1 [file Supplementary_file_1.docx]

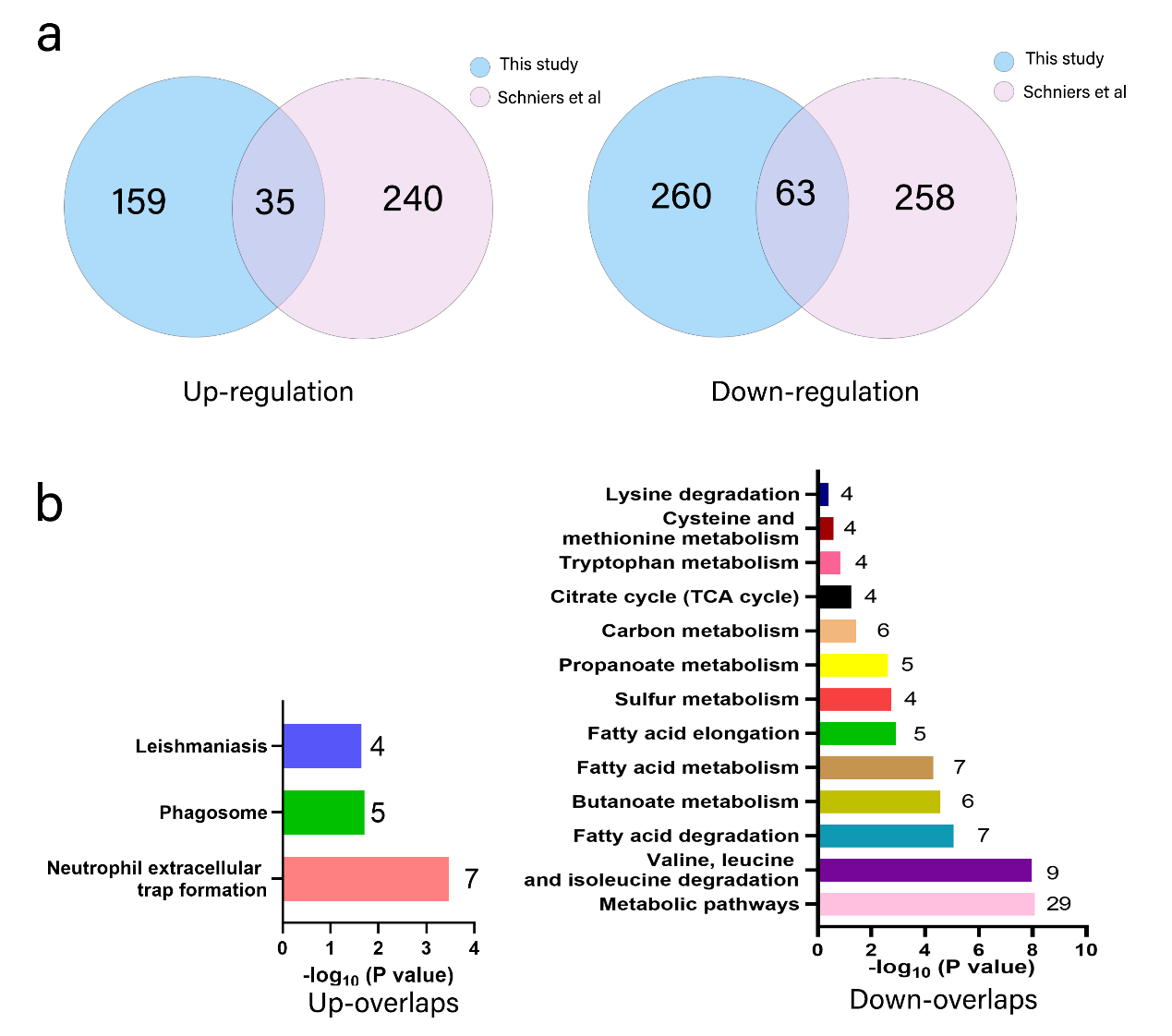


Figure S1. Comparative analysis based on protein characteristics in this study and Schniers et al. (2019). a) Venn diagram summarizing overlaps and differences between two studies. b) KEGG pathway analysis of overlaps proteins. The horizontal axis represents -log10(P value), the ordinate represents the KEGG pathway, and the numbers represent the number of differential genes enriched in the pathway.
